# Assessment of Dry Mouth in a Palliative Population: A Comparison Between Patient-Reported Symptoms and Clinical Oral Dryness Scale Measurements

**DOI:** 10.1177/10499091251356596

**Published:** 2025-06-30

**Authors:** Emir Murphy Dourieu, Dominika Lisiecka, William Evans, Patricia Sheahan

**Affiliations:** 1 Kerry Specialist Palliative Care Service, 57986 University Hospital Kerry, Tralee, Ireland; 2 Department of Nursing and Health Care Sciences, 8813 Munster Technological University – Kerry Campus, Tralee, Ireland; 3 Department of Medicine, University College Cork, Cork, Ireland

**Keywords:** xerostomia, dry mouth, palliative care, patient-reported measures, dry mouth assessment, oral dryness

## Abstract

The severity and impact of dry mouth in patients receiving palliative care is underreported, and assessment methods for this condition are lacking consensus. This study aimed to examine the severity of dry mouth in a palliative population as rated on a visual oral dryness scale and to compare these findings with the actual symptoms experienced by patients, as reported through both a symptom scale measure and a bother questionnaire. 40 participants took part in this cross-sectional study. The results indicated that xerostomia is a significant and debilitating symptom for many patients receiving palliative care. Daily activities such as speaking and sleeping were rated as highly affected by xerostomia, whereas swallowing was not reported to be as badly affected. The use of the oral dryness scale as an assessment technique in this population showed correlation with some, but not all, of xerostomia-related symptoms experienced by participants.

## Introduction

There is increasing recognition of the importance of healthy oral status, especially in people receiving cancer treatments and those who are critically ill or immunocompromised. The evidence reports that the oral microbiome is linked with body wellbeing,^1-3^ and soreness in the oral cavity tends to reduce oral intake, which further affects health outcomes.^4,5^ Oral care is an area which has typically been neglected due to many factors.^6-9^ These factors include limited understanding of the importance of oral care, lack of agreed oral hygiene protocols and poor education of staff, lack of access to specialists in oral hygiene, and low levels of staffing in health institutions.^6,8,10,11^

An important consequence of many medications and treatments in the palliative population (chemotherapy, radiation, immunotherapy) is the development of dry mouth. In a recent systematic review of oral health problems in this population dry mouth was the highest reported oral issue.^7^ Dry mouth can have many consequences which include difficulty eating and speaking, difficulty sleeping, excessive thirst, bad breath, dental issues, and increased risk of candida.^12-18^ Dry mouth can happen due to salivary gland hypofunction, and this can result in changes in both the quantity and quality of saliva.^18-20^

Saliva capturing techniques may be used to assess dry mouth.^21-26^ While these methods are useful in quantifying volume of saliva, they are not always available to use as a bedside clinical measure of dryness. Therefore, several scales have been developed as a more practical clinical measure of dry mouth.^27-29^ The term xerostomia is defined as a subjective sensation of dry mouth.^30,31^ While xerostomia has been well documented in conditions such as Sjogren’s disease and diabetes,^32-34^ research on the impact of xerostomia in palliative populations are few despite the high prevalence of this condition.^35^

Information about the severity and impact of xerostomia (perceived dryness of the mouth) on the daily lives of patients receiving palliative care is poorly documented and it is not known how xerostomia correlates with the objective measures of mouth dryness. This study therefore aims to examine the severity of dry mouth in a palliative population and compare objective severity ratings with the subjective symptom experiences. The ultimate goal is to enhance our understanding of dry mouth in a palliative population and how this dryness is assessed and perceived.

## Methods

This paper reports the design and findings of a cross-sectional study. Data were collected from medical charts, rating scales and oral examination of dry mouth using the Challacombe Oral Dryness scale (CODS).^28^ Subsequently, short qualitative interviews were conducted to understand the impact of xerostomia and these findings are reported elsewhere.^15^

### Setting

Participants were recruited from a Specialist Palliative Care Service in Southern Ireland (in-patients and out-patients).

### Recruitment and Population

Patients presenting with dry mouth were identified by staff of the palliative care unit (purposive sampling). Eligibility criteria are presented in Table 1. Phase of illness according to the Karnofsky Performance Scale (AKPS) was also noted to ensure that eligibility criteria of 30% or more was met. This determined that all patients were communicative, and well enough to participate in this study and were not at an end-of-life phase of their illness. Ethical approval for the study was obtained from the Clinical research Ethics Committee of the Cork Teaching Hospitals and from the Institute of Research Ethics Committee of Munster Technological University. The sample size planned for this study was 45 based on following input parameters, using G*Power 3:

(1) Using the Chi Square inferential procedure
(2) Effect size = .45
(3) Significance level α = .05
(4) Power (1-β) = .85

**Table 1.** Eligibility Criteria

**Inclusion criteria**
In patient or out-patient of local specialist palliative care service
>18 years
AKPS >30%
Verbal or non-verbal and able to provide informed consent
**Exclusion criteria**
Drowsiness
Excessive pain or distress (at baseline or due to research)
Medical contraindications - as per leading consultant
Temporomandibular joint issues or mouth opening <20 mm
Diagnosis of Sjogren’s
Cognitive impairment affecting consent or unable to consent
Non-proficient English

### Scales and Oral Examination

Two different scales were used to obtain quantitative data about the dryness of the mouth for each participant (during the same session). Both scales focused on the subjective rating of the symptom of dry mouth by the participant. The first scale used was the Palliative Care Outcome Collaboration (PCOC) Symptom Assessment Scale (SAS).^36^ This is a validated instrument used in the assessment of patients in palliative settings worldwide. The scale has subdivisions ranging from zero for absent, mild (1-3), moderate (4-7), severe (8-10). Participants were asked to rate their level of dry mouth on the scale at the time of interview and this data was recorded for later analysis.

The second scale was a modified Xerostomia Bother Questionnaire (based on the bother questionnaire printed by Saliva Orthana). This scale focused on the physical and functional symptoms associated with dry mouth and was used to obtain specific information about effects on taste, swallowing and speech,. This scale is a 5-point rating scale where 0 indicates not being bothered by a symptom, to 4 being severe. See Appendix 1 for a full example of the symptoms associated with xerostomia which were rated by each participant.

An oral examination of the mouth was conducted following the validated Challacombe Oral Dryness Scale (CODS).^28^ CODS is a ten-point scale rating saliva viscosity, oral debris and mucosal appearance. A 12 mm disposable plastic laryngeal mirror was used for the friction test. Along with the CODS scale, any other incidental but significant findings such as candida, tongue coating, or ulceration were also noted. Those who were agreeable had photographic images of their mouth taken (Supplemental Material 1). These were recorded using a standard iPad and images were shown to a consultant dentist who corroborated the CODS scale ratings.

Data were collected in 2019/2020 over a period of a year by the first author, a Clinical Specialist Speech & Language Therapist experienced in specialist palliative care.

## Results

A total of 52 participants were identified as eligible for the study but 12 were excluded for the following reasons: condition deterioration (n = 7), early discharge home (n = 1), cognitive impairment (n = 2), change of mind (n = 1) or dry mouth resolution (n = 1). The final sample was 40 (of which 34 were inpatient) (Table 2). We were unable to recruit more participants mainly due to a cyberattack on the local health system which occurred during the data collection. It resulted in reduced services (no out-patient clinic) due to lack of access to electronic records. Recruitment was also difficult due to Covid-19 pandemic, which necessitated maintaining social distance, use of personal protective equipment, and limited contact time (15 min).

**Table 2.** Participant Demographics

Age	Mean
	70.5 years
	Range
	38-92 years
Gender	Male (n = 20)
	Female (n = 20)
Service type	In patient (n = 34)
	Out patient (n = 6)
Participant diagnosis	Colorectal cancer (n = 8)
	Lung cancer (n = 6)
	Breast cancer (n = 5)
	Head & neck cancer (n = 3)
	Cholangio cancer (n = 3)
	Prostate cancer (n = 3)
	Ovarian cancer (n = 2)
	Myeloma (n = 2)
	Motor Neurone Disease (n = 2)
	Brian cancer (n = 1)
	Pancreatic cancer (n = 1)
	Renal failure (n = 1)
	Respiratory disease (n = 1)
	Spinal degeneration (n = 1)
	Primary unknown (n = 1)
AKPS level	40/50 (n = 25)
	60/70 (n = 14)
	80 + (n = 1)

## Medications

Medication list was extracted from medical files. The majority of participants were on ≥ 9 medications. The most commonly used medications were proton pump inhibitors, followed closely by opioids (see Appendix 2).

## Dental Status

82.5% of participants had full dentition either via natural teeth (42.5%), full denture (15%) or mixed teeth/partial denture (25%). The remaining participants had minimal teeth (10% ≤ 6 teeth) or were edentulous (7.5%).

## Severity Levels of Dry Mouth and Xerostomia

**Figure 1.**
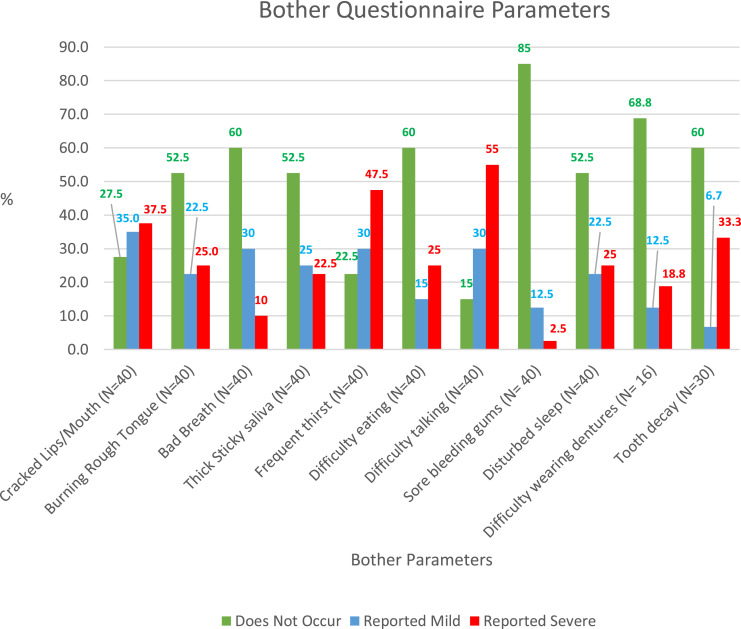
Bother Questionnaire Findings.

Severe xerostomia was reported by 30% of participants and a further 52.5% rated it as moderate. The outcomes of the objective assessment of dry mouth were different, with the majority scoring in the moderate range (25%), followed by mild (10%) and severe (5%). The most frequent rating overall using the Xerostomia Bother Questionnaire was difficulty talking, with 55% rating it as a severe occurrence and a total of 85% between moderate or severe (Figure 1). Thus, dry mouth was linked with difficulty talking to some degree in 85% of participants. Frequent thirst was rated as being severe in almost half of all participants (47.5%), with a further 30% rating as a mild symptom. In contrast, over 20% of participants reported no thirst despite having dry mouth.

Finally, with regard to severity levels, the mean score of all 11 parameters was calculated; the overall mean for all 11 parameters was 1.79 with the lowest value at 1.10 and the highest at 2.70. This indicates, on average, an approximate moderate perceived level of bother. Factors were also computed into 2 sub sections, specifically those that related to daily activities and those that related to physical effects. On the mean scores for these 2 categories there is little difference in terms of the severity of the effect on daily activities against physical effects. (Table 3).

**Table 3.** Bother Parameter Ratings

Bother parameter ratings	Mean	Standard deviation	Minim. Value	Maxim. Value
Overall mean of all 11 bother factors ratings (1-3)	1.79	.409	1.10	2.70
Mean of combined effect on daily activities Level of thirst, difficulty talking, difficulty eating, difficulty sleeping, and difficulty wearing dentures	1.96	.536	1.00	3.00
Mean of combined physical effects Cracked lips, burning rough tongue, bad breath, thick sticky saliva, tooth decay, sore bleeding gums	1.64	.435	1.00	2.67

## Impact of Dry Mouth – Physical Effects

Examination of specific oral symptoms resulted in a wide range of variation in symptoms. Bad breath emerged as an infrequent symptom as it was reported as absent in 60% of cases and only occurred mildly in 30% of cases. A burning rough tongue, or thick sticky saliva were both absent in over half of participants (52.5% for both symptoms). Both symptoms occurred severely in up to 1/4 of patients (25% and 22.5% prospectively). Cracked lips or corners of the mouth was reported as a more common symptom, with 35% reporting it happened on a mild basis and 37.5% reporting it as a severe problem (total of 72.5% experiencing it to some degree). Bleeding gums was the factor least reported with 85% stating that it never occurred and a further 12.5% stating that it was only a mild symptom. Tooth decay resulted in mixed findings with 60% reporting that it was not associated with dry mouth but of the remainder 33.3% listed it as a severe finding. In the 16 denture wearers, 68.8% reported no difficulty wearing dentures in the presence of dry mouth.

On oral examination, candida was rated as not present in 57.5%. In the remaining participants candida was either undetermined (20%), or candida was highly likely in 22.5%. Cases where candida was undetermined included participants with severe tongue coating which may or may not have been candida related. Angular cheilitis was obvious in only 1 participant.

The Xerostomia Bother Questionnaire parameters which relate to physical issues only (cracked lips, burning rough tongue, bad breath, thick sticky saliva, tooth decay, and sore bleeding gums) were grouped to create a mean score of physical effects of dry mouth. When compared with the dry mouth severity ratings, both subjective (SAS) and objective (CODS), statistically significant correlations were found, *r*_*s*_ = .550 , *P* < 0.01 and *r*_*s*_ = .467 , *P* < 0.01 respectively. Fisher's exact tests indicated no significant associations (Table 4).

**Table 4.** Correlations and Associations

Correlations and associations	Spearman’s r_s_ value^a^	Fisher's exact probability^b^	Cramer’s V
Correlation between subjective and objective dryness			
1. Overall subjective (SAS) and objective (CODS)	.424**	.016*	.357
2. Mean subjective (Bother) an objective (CODS)	.381*	-	-
Correlation between effect on communication and severity of dryness			
1. Difficulty talking and subjective dryness (SAS)	.437**	.019*	.383
2. Difficulty talking and objective (CODS)	.096	.979	-
Correlation between effect on eating and severity of dryness			
1. Difficulty eating and subjective dryness (SAS)	.416**	.103	-
2. Difficulty eating and objective dryness (CODS)	−.046	.936	-
Correlation between effect on daily activities (thirst, difficulty eating, difficulty talking, difficulty sleeping, difficulty wearing dentures) and severity of dryness			
1. Mean daily activities and subjective dryness (SAS)	.566**.	-	-
2. Mean daily activities and objective dryness (CODS)	.170	-	-
Correlation between physical effects (cracked lips, burning rough tongue, bad breath, thick sticky saliva, tooth decay, sore bleeding gums) and severity of dryness			
1. Mean physical effects and subjective dryness (SAS)	.550**	-	-
2. Mean physical effects and objective dryness (CODS)	.467**	-	-
Correlation between medication levels and severity of dryness			
1. Medications and subjective dryness (SAS)	.199	.606	-
2. Medications and objective level (CODS)	.149	0.9	-	

^a^rs Spearman’s Rank correlation

^b^ Fishers Exact probability

Significance of Correlations: †P < 0.100. *P < 0.050. **P < 0.010. ***P < 0.001

## Impact of Dry Mouth - Effects on Daily Activities of Talking, Eating, and Sleeping

Only 15% of cases stated that dry mouth had no effect on their ability to talk. This symptom was the most common effect of dry mouth among the participants studied. Using the Spearman’s rank test there was a strong positive correlation between difficulty talking and subjective dryness (SAS), *r*_*s*_ = .437 , *P* < 0.01. Fisher’s exact test was used to compare difficulty talking and subjective dryness (SAS). There was a statistically significant association between difficulty talking and subjective dryness (SAS), two-tailed *P* = .019. The association was strong (Cohen, 1988), Cramer’s V = .383. Of those respondents who reported mild difficulty talking (n = 12), 5 respondents reported mild dryness. Respondents reporting severe difficulty with talking (n = 22) had a similarity match of moderate dryness in 11 cases and severe dryness in 10 cases. No significant association was found between difficulty talking and the objective oral dryness measure (CODS) (Table 4).

With regard to swallowing and dry mouth, 60% of cases reported that it had no effect on swallowing and only 25% of cases rated it was having severe effect on swallowing. Using the Fisher’s exact test, no statistically significant association between difficulty eating and subjective (SAS) dryness was evident, (*P* = .103). In addition, no statistically significant association was found between difficulty swallowing/eating and the objective oral dryness score (CODS), two-tailed *P* = .936 (Table 4).

The effect of dry mouth on continued sleep resulted in a 50/50 split with 52.5% of participants stating that dry mouth had no effect on their sleep pattern and the remainder stating that it had some effect or tended to wake them at night. Of these, 25% rated this effect is severe.

Finally, statistical analysis using the mean score for activity-related parameters (thirst, difficulty eating, difficulty talking, difficulty sleeping, difficulty wearing dentures) was applied. A statistically significant correlation was found between the mean daily activity score and the subjective dry mouth scores (SAS), *r*_*s*_ = .566 , *P* < 0.001. No statistically significant correlation was found between the mean daily activity score and the objective oral dryness score (CODS), *P* = .170 (Table 4).

## Discussion

The results of this study indicate that xerostomia is present and problematic for the many patients receiving palliative care. The overall level of xerostomia, reported as moderate to severe in 82.5% of the group, is comparable to the findings of several published studies.^13,37^ Xerostomia that is reported using the SAS scale correlates with oral examinations of visual dryness observed in the mouth using the CODS scale, although patients’ experiences of dry mouth can be worse than visual evidence suggests. In clinical settings the findings of this study indicate that observations of the mouth alone are not adequate to ensure effective evaluation of dry mouth as the symptoms experienced and effects on various activities (speaking, eating, etc.), may be significantly worse than visual observations. There is a need to ensure that additional questions or scales are completed to provide the information necessary to rate the actual effect that the dry mouth is having on a patient’s daily life. The SAS scale is a global symptom measurement and as such does not have the capacity to rate the frequency or persistence of dryness over time. There is a need therefore to ensure that adequate discussion about xerostomia is taking place for patients receiving palliative care. A symptom scale, such as the MSAS, which rates a symptom according to frequency, intensity, distress, and need for treatment,^38^ or the Oral Symptom Assessment Scale – OSAS,^39^ may yield more detailed and helpful information regarding xerostomia characteristics.

Findings of the Xerostomia Bother Questionnaire suggest that difficulty with speech, frequency of thirst, and cracked lips rated highly as a concern for participants with dry mouth. The viscosity of saliva also generates interesting findings. Closer examination of the 12 participants who rated overall xerostomia as severe, reveals that 84% referred to the issue of thick sticky mucous as being a severely debilitating effect of dry mouth. Previous quantitative studies described in the literature review do not appear to capture this effect when discussing severity of oral conditions and dry mouth in patients receiving palliative care although a few qualitative studies refer to “viscous ropey secretions” or “viscous slime” as a finding reported by care staff.^10,40^

Using the CODS scale clinically is complicated by the presence of candida or tongue coating. These white or red lesions can make the rating of tongue fissuring or tongue papillae more difficult to view as they mask the underlying mucosa. The finding of candida in the study was not negligible as it was obviously present in 22.5% of participants or “undetermined” in a further 20%. Definitively ruling out its presence in cases of dry mouth based on visual evaluation alone is difficult. Previous studies of patients receiving palliative care seem to support this very difficult area of reporting as they vary greatly in their findings.^12,41,42^ In a systematic review of the most frequent oral conditions in patients in palliative care, candida is listed as one of the top three oral conditions along with dry mouth and dysphagia.^11^ Detailed studies about candida and tongue coating in palliative populations are lacking and possible confusion between tongue coating and candida may be occurring. The use of improved diagnostic testing for this condition may need to be considered.

One of the most significant findings of this study is the very high levels of reported difficulty with talking associated with dry mouth. With 85% of the 40 participants reporting at least some difficulty with it and 55% reporting it as severe, this dry mouth effect needs careful monitoring. The positive association of talking with overall SAS level also suggests that as severity of xerostomia increases there are increased perceived effects on speech. It is interesting that so few previous studies regarding dry mouth make reference to the effect on speech. While some studies^43-45^ refer to issues with voice occurring when dry mouth is severe, few draw attention to speech effects.^13,46^

Only a quarter of the group studied reported dry mouth having a severe effect on eating and drinking and results indicate that the process of swallowing is not reported as problematic at all in over half of participants. Comparison with previous studies of palliative populations referring to dysphagia, is complex as dry mouth is often not the only factor under examination. Variability from 15% to 56% have been noted in previous studies,^42,47^ of palliative populations but these variances may relate to the actual sample chosen in each study. The level of oral residue has been shown to increase with increased severity of dry mouth,^43^ but many participants of this study report that they reduce any dysphagia effects by drinking more water and washing food down.

The inherent value of this study is that it clearly identifies some of the complexity of assessing dry mouth and it indicates where assessment may be falling down. Our data confirms that there is a need to combine both observational/objective and self-reported elements in any assessment of dry mouth in the palliative setting. Furthermore, there is a need to ensure that specific questions which pertain to quality of life are included as part of any dry mouth assessment as ratings on a single scale alone, such as the SAS, while useful for monitoring change, will not provide the in-depth information necessary to establish ongoing physical or psychological effects and are unlikely to guide management. Ensuring that patients are listened to with regard to speech, swallowing, taste effects and previously tried strategies is likely to be much more beneficial in terms of management.

## Strengths and Limitations

The generalizability of the results is limited owing to the sampling method, composition and small sample size. The quality of the photographic images used in this study are not equal to those obtained using dental imaging techniques, as they were obtained using an iPad camera. In addition, the social contact restrictions during Covid-19 pandemic necessitated rapid imaging. Future studies would benefit from using specialist intra-oral equipment. The images achieved were a significant addition to the overall study as it is rare to have data where patient reports are linked with photographic images.

The CODS scale has been successfully used and demonstrated to have excellent reliability by other studies.^48-50^ Therefore it meets the criteria for suitability in research and is therefore a strength of this study. Use of a xerostomia questionnaire such as the XI may be suggested for future studies as it carries strong validity.^29^ Furthermore, a bother questionnaire such as the BI would allow improved accuracy with regard to timing and constancy of dry mouth. In contrast, the Xerostomia Bother Questionnaire used in this study, provided information about areas such as sleep, thirst and speech. This information could not have been achieved with pre-existing questionnaires at the time. However, the Oral Symptom Assessment Scale (OSAS), validated in the population of people with advanced cancer, may now provide this type of information.^39^

Finally, regarding the levels of severity as determined by the CODS, the effects of circadian rhythms on saliva production also needs to be considered as not all participants were seen at the same time of day.^51^ Furthermore, the timing effects of certain saliva-reducing medications may have affected results. It is evident from this study that patients in palliative settings are on large a number of drugs and in particular, the saliva drying effects of PPIs, opioids, steroids, benzodiazepines, and anti-depressants needs to be considered. There may be a need to complete a risk/benefit type analysis with regard to medication,^52^ to ensure that the control of particular symptoms are not outweighed by the disadvantage of salivary gland hypofunction or xerostomia.

Overall, the research design of this study is appropriate for the palliative population and the above limitations do not indicate that the results are not valid. On the contrary, the quantitative results have valid inferences determined by relevant statistical tests. Future research may enhance these findings through attention to the detail provided about limitations.

## Supplemental Material

Supplemental Material - Assessment of Dry Mouth in a Palliative Population: A Comparison Between Patient-Reported Symptoms and Clinical Oral Dryness Scale Measurements Supplemental Material for Assessment of Dry Mouth in a Palliative Population: A Comparison Between Patient-Reported Symptoms and Clinical Oral Dryness Scale Measurements by Emir Murphy Dourieu, Dominika Lisiecka, William Evans and Patricia Sheahan in American Journal of Hospice and Palliative Medicine®

## Appendix

**Appendix 1. Xerostomia Bother Symptom List:** Xerostomia Bother Symptom List

	Symptom rated 0 – 4
1	Dry cracked lips
2	Cracked corners of the mouth
3	Burning rough tongue
4	Halitosis (bad breath)
5	Thick sticky saliva in the corners of the mouth
6	Frequent thirst
7 (A) 7 (B)	Difficulty talking Difficulty eating and swallowing
8	Increase in plaque and/or decay
9	Sore bleeding gums
10	Difficulty wearing dentures
11	Disturbed sleep

Saliva Orthana (2018).

**Appendix 2.** Xerostomia Medications List

Type of drug known to induce xerostomia	N	%
Proton pump Inhibitor	30	75.0
Opioid	28	70.0
Steroid	22	55.0
Benzodiazepine	20	50.0
Anti-depressant	20	50.0
Anti-epileptic	18	45.0
Anti-cholinergic	11	27.5
Anti-psychotic	9	22.5
NSAID	7	17.5
Ace Inhibitor	7	17.5
Anti-histamine	7	17.5
Anti-microbial	7	17.5
Diuretic	6	15.0
Betablocker	4	10.0
H2 Antagonist	2	5.0
Anti-coagulant	2	5.0
Leukotrine Receptor Agonist	2	5.0
Anti-diabetic	1	2.5
Immunosuppressant	1	2.5
Alpha blocker	1	2.5
Calcium antagonist	1	2.5
Anti-emetic	11	2.5
Opioid antagonist	1	2.5
Lipid lowering	1	2.5
