# Supplementary material for: Assessment of Dry Mouth in a Palliative Population: A Comparison Between Patient-Reported Symptoms and Clinical Oral Dryness Scale Measurements
Source: Am J Hosp Palliat Care. 2025 Jun 30;43(8):833–41. doi: 10.1177/10499091251356596 (PMC13254131; doi:10.1177/10499091251356596)
Supplement: Supplemental Material - Assessment of Dry Mouth in a Palliative Population: A Comparison Between Patient-Reported Symptoms and Clinical Oral Dryness Scale Measurements [file sj-pdf-1-ajh-10.1177_10499091251356596.pdf]

Supplementary material 1

Cracked Lips (P9)<br>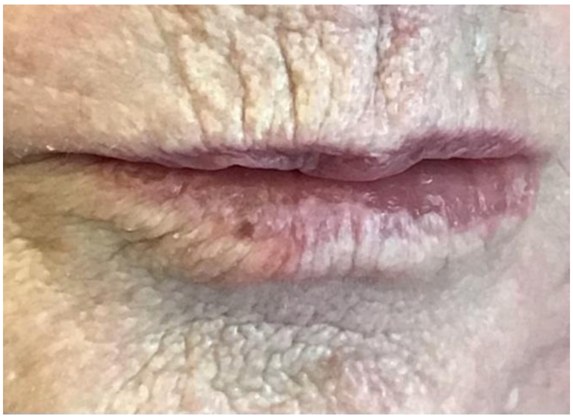	Tongue coating (P24)<br>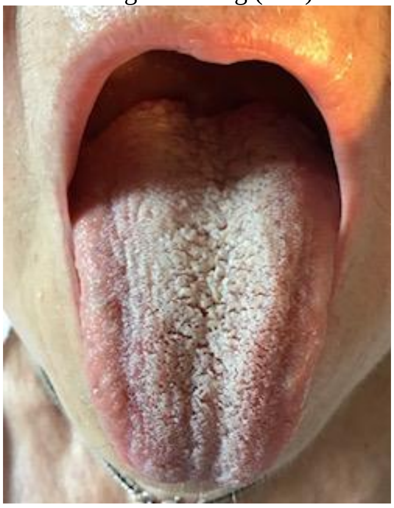
Debris stuck in teeth (P19)<br>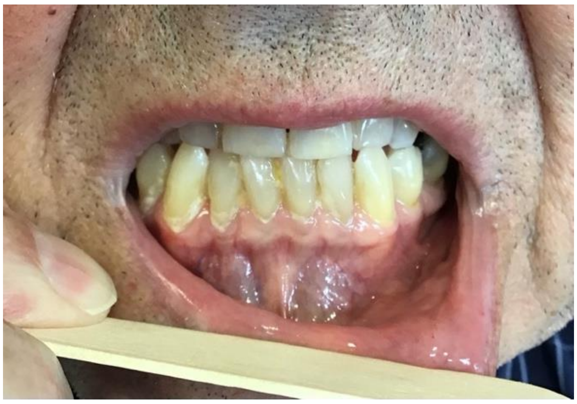	Loss of papillae (P15)<br>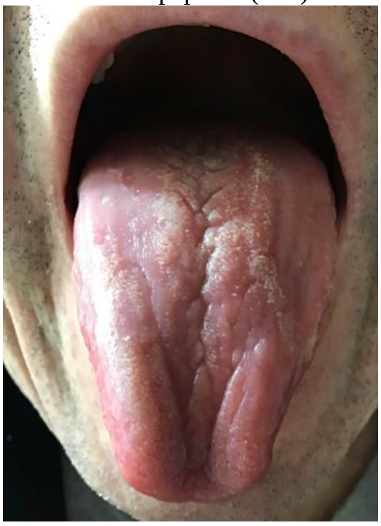
Angular Cheilitis (P40)<br>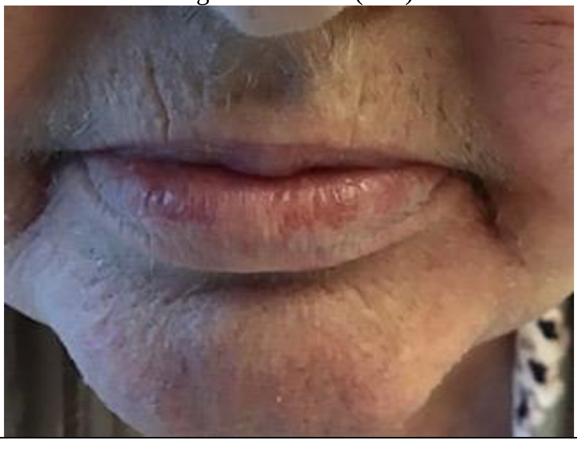	Dry fissured tongue (P30)<br>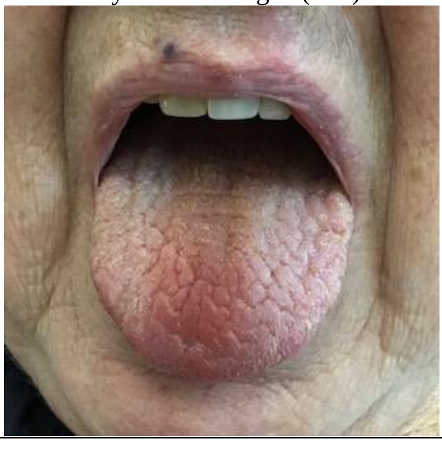
